# Preparation of Thermosensitive Gel for Controlled Release of Levofloxacin and Their Application in the Treatment of Multidrug-Resistant Bacteria

**DOI:** 10.1155/2016/9702129

**Published:** 2016-09-05

**Authors:** Danilo Antonini Alves, Daisy Machado, Adriana Melo, Rafaella Fabiana Carneiro Pereira, Patrícia Severino, Luciana Maria de Hollanda, Daniele Ribeiro Araújo, Marcelo Lancellotti

**Affiliations:** ^1^Laboratory of Biotechnology (LABIOTEC), Institute of Biology, University of Campinas (UNICAMP), Campinas, SP, Brazil; ^2^Laboratory of Nanotechnology and Nanomedicine (LNMed), Institute of Technology and Research (ITP), Aracaju, SE, Brazil; ^3^Tiradentes University (UNIT), Aracaju, SE, Brazil; ^4^Center of Natural Sciences and Humanities, Federal University of ABC (UFABC), Santo André, SP, Brazil; ^5^Faculty of Pharmaceutical Sciences, University of Campinas (UNICAMP), Campinas, SP, Brazil

## Abstract

Levofloxacin is a synthetic broad-spectrum antibacterial agent for oral or intravenous administration. Chemically, levofloxacin is the levorotatory isomer (L-isomer) of racemate ofloxacin, a fluoroquinolone antibacterial agent. Quinolone derivatives rapidly and specifically inhibit the synthesis of bacterial DNA. Levofloxacin has* in vitro* activity against a broad range of aerobic and anaerobic Gram-positive and Gram-negative bacteria. However, formulation of combined poloxamers thermoregulated (as Pluronic® F127) and levofloxacin for use in multiresistant bacterial treatment were poorly described in the current literature. Thus, the aim of the present work is to characterize poloxamers for levofloxacin controlled release and their use in the treatment of multidrug bacterial resistance. Micelles were produced in colloidal dispersions, with a diameter between 5 and 100 nm, which form spontaneously from amphiphilic molecules under certain conditions as concentration and temperature. Encapsulation of levofloxacin into nanospheres showed efficiency and enhancement of antimicrobial activity against* Escherichia coli*,* Pseudomonas aeruginosa*, and* Klebsiella pneumoniae* when compared with only levofloxacin. Furthermore, all formulations were not cytotoxic for NIH/3T3 cell lineage. In conclusion, poloxamers combined with levofloxacin have shown promising results, better than alone, decreasing the minimal inhibitory concentration of the studied bacterial multiresistance strains. In the future, this new formulation will be used after being tested in animal models in patients with resistant bacterial strains.

## 1. Introduction

In recent years, bacterial resistant infections have become a global health challenge and are threatening the health of societies [1–4]. Due to the emergence of resistant infections, existing antibacterial drugs have become less effective or even ineffective; this has led to the development of new antibacterial drugs [1]. Also, quinolones are one of the most commonly prescribed classes of antibacterials in the world and are used to treat a variety of bacterial infections in humans [5].

The founding member of the quinolone drug class, nalidixic acid, is a naphthyridine that was first isolated by George Lesher and colleagues in 1962 as a byproduct of chloroquine synthesis [5, 6]. Nalidixic acid was introduced into the clinic in the 1960s for the treatment of uncomplicated urinary tract infections caused by enteric bacteria [5, 7]. By the 1970s, several first-generation quinolones, oxolinic acid being the most notable, had been synthesized and introduced into the clinic [5, 7–10].

Quinolones were a little-used drug class until the early 1980s, when a second generation of compounds was developed [5, 7–10]. These newer drugs, highlighted by norfloxacin, ciprofloxacin, and ofloxacin, displayed considerably improved activity against gyrase, greater penetration into Gram-positive organisms, and enhanced pharmacokinetics and pharmacodynamics [5]. The most critical changes to the quinolone skeleton were the introduction of a fluorine at position C6 and a major ring substituent (piperazine or methylpiperazine) at C7 [5, 7–10]. Because of the inclusion of the fluorine, quinolones are often termed “fluoroquinolones” [5].

Norfloxacin is considered to be the first broad-spectrum quinolone and was utilized to a far greater extent than nalidixic acid [5, 7–10]. Unfortunately, because of low serum levels and poor tissue penetration, norfloxacin was still confined to use for the treatment of urinary tract infections and sexually transmitted diseases [5]. Ciprofloxacin was the first quinolone that displayed significant activity outside of the urinary tract [5, 7–10]. After more than 20 years in clinical use, ciprofloxacin remains one of the most commonly prescribed antibacterial drugs and is used to treat a variety of Gram-negative and, to a lesser extent, Gram-positive infections [11–13].

The clinical success of ciprofloxacin spawned an array of newer-generation quinolones that displayed an even broader spectrum of activity, especially against Gram-positive species [5, 7–10]. Levofloxacin, moxifloxacin, and sparfloxacin have enjoyed the most success and display good activity against Gram-positive respiratory tract infections. Furthermore, the pharmacokinetics of levofloxacin are advantageous compared to those of other members of the drug class, and treatment requires only a single pill per day [5, 14, 15].

Levofloxacin is a member of the fluoroquinolone class of antibacterial agents with microbiological activity against clinically relevant bacteria that cause respiratory, skin and skin structure, and genitourinary tract infections [15]. Chemically, this antibiotic is a chiral fluorinated carboxyquinolone, which is the pure (−)-(S)-enantiomer of the racemic drug substance ofloxacin.

The chemical name is (−)-(S)-9-fluoro-2,3-dihydro-3-methyl-10-(4-methyl-1-piperazinyl)-7-oxo-7H-pyrido[1,2,3-de]-1,4-benzoxazine-6-carboxylic acid hemihydrate [16]. The mechanism of action of levofloxacin and other fluoroquinolones antibacterial complexes interferes with bacterial DNA metabolism by inhibiting two bacterial enzymes, DNA gyrase and topoisomerase IV, which are critical to DNA replication, transcription, repair, and recombination [15]. Their primary targets against Gram-positive and Gram-negative bacteria are bacterial topoisomerase IV and DNA gyrase, respectively [15, 17].

Not surprisingly, the number of quinolone-resistant bacteria is rising. This can occur during handling, especially with* Pseudomonas* infection, which is more common if the patient previously received drugs and in blood with nonenough dose for winner infection [18, 19]. For* S. aureus* and* P. aeruginosa*, a single mutation is enough. More than one mutation is needed for* E. coli* to become resistant to quinolone [20, 21].

Due to the development of antibiotic resistance and the outbreak of infectious diseases caused by resistant pathogenic bacteria, pharmaceutical companies and researchers are now searching for new unconventional antibacterial agents. The demand for individualized therapy and lower risks of adverse effects has always been a goal for health professionals. Besides, new pharmaceutical formulations seeking to increase efficiency and reduce drug toxicity are currently being researched [22].

Polymers are the substances of high molecular weight having repeating monomer units [23]. They are widely used in pharmaceutical systems as suspending, adjuvants, adhesives, emulsifying agents, and coating material for controlled and site specific drug delivery systems [23]. Polymer molecules may be branched or linear and separate linear or branched chains may be joined by crosslinks [23]. The chemical reactivity of polymers depends upon the chemistry of monomer units but their properties depend to a large extent on the way of arrangement of the monomers [23]. Polymers having identical monomeric units are referred to as homopolymers; those formed from more than one monomer type are called copolymers [23]. Arrangements of various monomers units, say A and B, lead to formation of varieties of copolymers [23]. The copolymers may be described as alternating copolymers, graft copolymers, or block copolymers [23]. Pluronic is one of the most widely used block copolymers and forms heterogels [23–25].

Poloxamers are interesting copolymers as a nanocarrier having amphiphilic characters [23]. Due to large solubility differences between hydrophobic and hydrophilic moieties, in aqueous medium, they are able to self-assemble into polymeric micelles characterized by mesoscopic size range [23]. These micelles consist of water-insoluble cores and water-soluble shells [23]. Depending on blocks length, the core can assemble into various supramolecular structures characterized by different morphologies [23, 26–28].

Currently, the potential applications of nanoparticles in advanced materials are being explored and are considered a key technology for the future [12, 29–31]. In this work, micelles were produced in colloidal dispersions using poloxamers, with a diameter between 5 and 100 nm, which form spontaneously from amphiphilic molecules under certain conditions as concentration and temperature [32, 33]. Due to their small size (<100 nm), the micellar carriers have a high penetration capacity in different tissues such as vascular endothelium and oral, nasal, and ocular mucosa [34]. Among the constituents of micellar systems, those of the block copolymer class of poloxamers stand out (Lutrol®, Pluronic, Synperonic®, and Tetronic®). These units determine the amphiphilic character of these molecules characterizing them also for different hydrophilic-lipophilic balance [35]. Furthermore, we investigated for the first time the effect of encapsulation of levofloxacin for controlled release and we verify the effectiveness of this process in the resistance of multidrug bacterial strains.

## 2. Material and Methods

### 2.1. Formulation of Combined Poloxamers Thermoregulated

The Pluronic F 108NF Prill Poloxamer 338 (PL 338, molecular weight: 14,600 Da), the Pluronic F 127NF Prill Poloxamer 407 (PL 407, molecular weight: 12,600 Da), and the Pluronic L-81 (L-81, molecular weight: 2,750 Da) were products of BASF Corp. (Ludwigshafen am Rhein, Germany) purchased from Sigma-Aldrich (St. Louis, MO, USA). Levofloxacin (analytical reference material, molecular weight: 361.37 Da), methylthiazolyldiphenyl-tetrazolium bromide (MTT, molecular weight: 414.32), Brain Heart Infusion Agar (BHI Agar), and Mueller Hinton Broth were purchased from Accumedia (Neogen Corporation, Lansing, MI, USA). RPMI-1640 medium, fetal bovine serum, antibiotic/antimycotic solution (with 100 UI·mL^−1^ penicillin and 100 *μ*g·mL^−1^streptomycin sulfate), PBS 1x with antibiotic/antimycotic solution, and Trypsin-EDTA were purchased from Cultilab Laboratory Materials for Growing Mobile (Campinas, São Paulo, Brazil). All other reagents were of analytical grade. Deionized water (Purelab Option-Q, ELGA LabWater, High Wycombe, UK) was used for all experiments [36].

### 2.2. Production of Polymeric Nanoparticle with or without Levofloxacin

Polymeric nanoparticle with or without levofloxacin was prepared in accordance with the cold method described by Schmolka [36, 37]. Before the incorporation of levofloxacin in the polymeric nanoparticle, different solutions containing PL 407 (Formulation F3, Table 1) and PL 338 (Formulation F4, Table 1) alone or in binary systems with L-81 (Formulations F1 and F2, Table 1) were dispersed in deionized water for 3 hours at 4°C under magnetic stirring (100 rpm). After complete dissolution of poloxamers in deionized water, levofloxacin (0.1 mg·mL^−1^) was dispersed in some formulations (F1-L, F2-L, F3-L, and F4-L, Table 1) for 3 hours at 4°C under magnetic stirring (100 rpm) [36, 37].

### 2.3. Size, Polydispersity Index, and Zeta Potential Analyses

Dynamic light scattering was performed according to dos Santos et al. [36]. Dynamic light scattering was used to determine the polymeric diameter, polydispersity index, and zeta potential. Measurements were performed using a particle analyzer, ZetaSizer Nano ZS (Malvern, UK), at a fixed angle of 173° and temperatures of 25°C and 37°C [36]. All formulations were prepared in deionized water and were filtered using a polycarbonate membrane (pore 0.22 *μ*m). Zeta potential values for all formulations were also measured in deionized water adjusting conductivity (50 *μ*S·cm^−1^) and were calculated from the electrophoretic mobility using the Helmholtz-Smoluchowski equation. The analyses were performed using the software included in the system [13]. All experiments were executed in triplicate.

### 2.4. Permeation Experiments

Permeation studies of levofloxacin and levofloxacin-loaded polymeric micelles were carried out in Franz diffusion cells (DISA, Milan, Italy) with 0.6 cm^2^ permeation area and a receiver compartment of 4.2 mL in volume. The mucosa was mimicked over a 0.45 mm cellulose filter (connective side of tissue facing the membrane filter) because of its fragility, avoiding any damage that could alter permeation parameters without altering levofloxacin transport. F1-L, F2-L, F3-L, and F4-L were applied in infinite dose conditions (1.76 g·cm^−2^) in the donor compartment. The receptor chambers were filled with NaCl 154 mM solution containing HEPES 20 mM, pH 7.4, magnetically stirred at 37°C.

Permeation experiments were performed in no occlusive conditions during a period of 24 hrs. Samples (1 mL) were periodically withdrawn from the receptor phase, analyzed by spectrophotometer, and replaced with fresh receptor solution in equal volumes. The flux of drug was calculated from the slope of the linear portion of the curve (cumulative amounts of levofloxacin transported across the mucosa per unit of area × time). Lag time was obtained from the interception to the time axis. The permeability coefficient was calculated according to [38]

(1) $$\begin{array}{c} J=P\times C_{d}, \end{array}$$

where* J *(*μ*g·cm^−2^·h^−1^) is levofloxacin flux across the skin,* P *(cm·h^−1^) is the permeability coefficient, and *C*_*d*_ is levofloxacin concentration in the donor compartment (*μ*g·cm^−3^).

### 2.5. Biological* In Vitro* Assays

#### 2.5.1. Antimicrobial Susceptibility Testing

An* in vitro *test using bacterial Gram-positive and Gram-negative strains was performed (Table 2). The antibacterial activity was initially determined using the double layer agar diffusion method and then the minimum inhibitory concentration (MIC) was determined for susceptible bacteria. Some resistant bacterial strains used in this work were kindly donated by Professor Dr. Ana Lúcia da Costa Darini (Faculty of Pharmaceutical Sciences of Ribeirão Preto, São Paulo, Brazil).

#### 2.5.2. Microdilution Test to Determine the Minimum Inhibitory Concentration (MIC)

Stock solutions of each formulation were prepared before the experiment. The final concentrations of these formulations tested ranged from 0.15 to 80 *μ*g·mL^−1^. This experiment was realized in 96-well plate and all analyses were based on the Fifteenth Informational Supplement of the Clinical Laboratory [39, 40] and Standards Institute.

Before the MIC experiment, the strains of interest were inoculated into BHI agar and incubated at 37°C for 16–24 h [41, 42]. On the next day, three to five well-isolated colonies of the same morphological type were collected and resuspended in 1 mL of sterile saline (0.9%) until final concentration of 1.5 × 10^8^ colony-forming units (CFU) mL^−1^ (0.5 McFarland standard, Probac, São Paulo, Brazil). Then, the bacterial suspension was diluted in each well of 96-well plate to a final concentration per well of 1 × 10^5^ CFU·mL^−1^ [39].

Next, the dilutions of all concentrations of all formulations were added in 96-well plate to a final volume of 0.1 mL. The positive (levofloxacin) control was prepared to a final volume 0.1 mL with 1 × 10^5^ CFU·mL^−1^ bacteria. Negative control was prepared with Mueller Hinton Broth without bacteria. The 96-well plate was incubated in ambient air at 37°C for 16–20 h. This experiment was run in triplicate for each strain and for controls [39].

### 2.6. Cytotoxic Activity

#### 2.6.1. Cell Culture

We purchased mouse fibroblasts NIH/3T3 (ATCC® CRL-1658*™*) cell lines from American Type Culture Collection (ATCC) CCL-1658. We chose the NIH/3T3 cells because they are widely used as an* in vitro* model for a tissue model. Therefore, if the material tested is cytotoxic on this cell, its use in therapy is discouraged [43–45]. This cellular lineage was grown in plastic flasks (25 cm^2^) with RPMI 1640 medium, supplemented with 10% inactivated fetal bovine serum and 1% antibiotic/antimycotic solution [43]. The cultures were incubated at 37°C in an atmosphere containing 5% CO_2_ [43]. Medium was changed every 48 h, and when the culture reached confluence, the subculture was treated with Trypsin-EDTA, until complete release of the cells. The released cells were transferred to a new plastic flask or 96-well plates [43–45].

#### 2.6.2. MTT Reduction Assay [43]

Before testing, we dispersed different concentrations of all formulations in culture medium without fetal bovine serum and antibiotic/antimycotic solution. We plated NIH/3T3 cells on 96-well plates [43–45]. We used 1 × 10^5^ cells·mL^−1^ to 1 × 10^7^ cells·mL^−1^ per well [43]. After this, we incubated the plate at 37°C, in a humidified incubator with 5% CO_2_, for 24 h [43]. Then, the medium was replaced and the test repeated with different formulations concentrations in the range between 5 and 200 *μ*g·mL^−1^, which was added to the wells in triplicate for each concentration. We used DL50 to define the CCN cytotoxicity (50% of the cells died) [43–45].

After 24 h of incubation, we removed the medium containing the formulations and washed each well 3 times with 0.1 mL of PBS Buffer 1x (137 mM NaCl, 10 mM phosphate, and 2.7 mM KCl, at pH of 7.4) [43]. Next, 0.2 mL of RPMI 1640 medium (without FBS and antibiotic/antimycotic solution) containing the dye MTT (0.5 mg·mL^−1^) was added [43, 44]. After incubation for 3 h at 37°C, we removed the medium with dye and carefully added 0.2 mL of ethanol to solubilize the blue formazan (yielded from MTT reduction by viable cells) [43, 45]. The plates were shaken for 10 min, and the absorbance for each well was read in a spectrophotometer, ELx800 Absorbance Microplate Reader (BioTek, USA), at *λ* = 570 nm. The values were expressed as percentages of MTT reduction compared to the control, where cells were not exposed to test agents [43, 46, 47].

### 2.7. Statistical Evaluation

Statistical analysis was performed using GraphPad Prism version 4.03. All experiments were conducted in triplicate and the results were expressed as the means ± standard deviation (SD). Data from each assay were analyzed statistically by ANOVA. Multiple comparisons among groups were determined with the Tukey test. Differences were considered significant when the *p* value was less than 0.05 [43–45].

## 3. Results and Discussion

Antibiotics are among the most important tools in medicine, but their efficacy is threatened by the evolution of resistance [11]. Since the earliest days of antibiotics, resistance has been observed and recognized as a threat; today, many first-generation drugs are all but ineffective [11]. We have thus far avoided a crisis through the continued modification of existing compounds and the discovery of new antibiotic classes [11] as well as the use of nanoparticles to engage and load this antibiotic, within this class of new antibiotics that include levofloxacin.

Levofloxacin is a broad-spectrum antibiotic of the fluoroquinolone drug class, mainly acting against Gram-positive and atypical agents [48, 49]. This antibiotic works as an inhibitor of DNA gyrase and topoisomerase IV causing bacterial death [50]. Despite their effectiveness, these antibiotics are commonly associated with undesirable side effects [50–52], mainly due to a tendency to aromatic stack among themselves under physiological conditions that reduce their bioavailability and become toxic [50, 53]. In this sense, encapsulation in smart nano- and microbiopolymeric devices is a novel technology potentially useful to provide effective controlled release of the drugs, therefore reducing their toxic concentrations [50]. Despite these, poloxamers were used in the nanoencapsulation of levofloxacin.

Among the several delivery systems reported in the literature, poloxamer- (PL-) based thermoreversible hydrogels have presented promising results in terms of improvement in the biopharmaceutical, pharmacodynamic, and pharmacokinetic properties of the incorporated drugs [36].

PLs (Pluronic) are copolymers consisting of polyethylene oxide (PEO) and polyoxypropylene oxide (PPO) units arranged in a basic structure A–B–A or PEO–PPO–PEO type [36]. Due to differences in the number of PPO and PEO units, PL monomers have the ability to self-assemble in micelles, presenting a hydrophobic core surrounded by a hydrophilic corona [35, 36]. At low temperatures, both PEO and PPO units are soluble in water [36]. However, when the temperature rises, the PPO units are dehydrated and aggregate (micellar core), while the hydrophilic PEO units (micellar corona) remain hydrated [36]. Subsequently, these micelles are assembled in different supramolecular structures such as hexagonal, cubic-ordered phases forming the hydrogels' organization phases [36]. This reversible phenomenon is characterized by a temperature range of sol-gel transition (*T*_sol-gel_), since, below this temperature, the systems remain as fluids and favor the development of injectable formulations but remain as semisolids close to the physiological temperature [36, 54–56].

These features associated with the biocompatibility of PL are essential for the development of delivery systems [36] for treatment of multidrug-resistant bacteria. In addition, the use of PL with different hydrophilic-lipophilic balance (HLB) [36] such as PL 407 and PL 403, PL 407 and PL L-81, PL 108 and PL 403, and PL 108 and PL L-81 as binary systems can be an interesting alternative to modulate the biopharmaceutical profile of these formulations [13, 36, 57–59].

The use of PL has been reported in the literature specifically for diverse types of treatment [36]. Previous studies showed the increased permeation of lidocaine and ketoprofen incorporated into PL 407 liposomal hydrogels, across the swine dural membrane [36, 60–62]. Subsequently, other studies presented the preparation and characterization processes of PL 407 hydrogels containing diclofenac [36, 63] and lidocaine [36, 64–66] as well as their pharmacological evaluations in animal models [36, 65, 67, 68] and humans [36, 69, 70].

However, these formulations were addressed for the use of a single type of PL. Moreover, there are no studies on PL-based* in situ* thermogelling hydrogel formulations containing levofloxacin. Therefore, in this work, we demonstrated for the first time PL 407 and PL 338 single-type loading levofloxacin and binary hydrogels composed of PL 407 and PL L-81, PL 338 and PL L-81, PL 108 and PL 403, and PL 108 and PL L-81 loading levofloxacin, reinforcing the importance of this work in the treatment of resistant bacterial infections, principally those in skin or exposed tissues.

**Figure 1:**
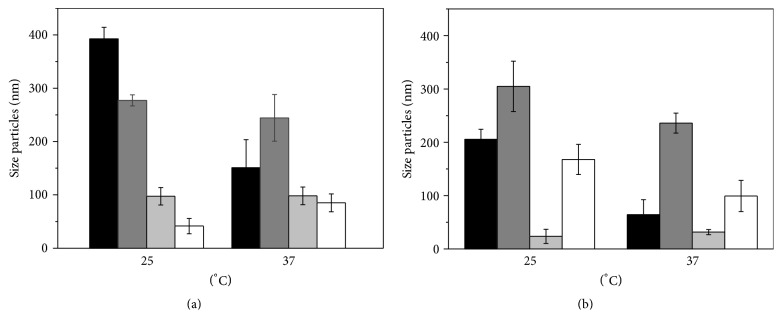
Characteristics of formulations in 25°C and 37°C: (a) polymeric micelles and (b) fluoroquinolone-loaded polymeric micelles.

The particle size ranged from 35 to 380 nm. In Formulations F1 and F2 with and F2 and F4 without levofloxacin, they decreased in size with increasing temperature. It is suggested that the increase in temperature caused rearrangement of the surfactant molecules compacting the micelles. However, in F5, temperatures of 37°C resulted in an increase in particle size in all cases. In Formulation 4 the preparation without levofloxacin showed a decline in nanoparticle size (Figure 1).

Table 3 presents the micellar mean, hydrodynamic diameter, the mean distribution, and zeta potential for the different systems composed of PL 407 or PL 338 isolated or in association with PL L-81 at room temperature (25°C) and body temperature (37°C).

At 25°C, the micellar systems composed of PL 407 and PL 338 presented a bimodal distribution, as observed by the two micellar diameters with ~5-6 nm (relative to the presence of PL unimers in solution) and ~40–50 nm. However, the hydrodynamic diameter analysis, at 37°C, showed a unimodal distribution, as a result of the micellar thermoresponsive self-assemblage observed for PL copolymers, considering the low polydispersion index (PDI = 0.10–0.12) values. Also, the presence of levofloxacin (LVF) into the systems changed the micellar dimensions, shifting the hydrodynamic diameter (~350–500 nm) to higher values and reducing zeta potential (−2 to −16 mV), for both temperatures, indicating the possible insertion of the drug into the micellar system. A similar pattern was also viewed after the insertion of PL L-81 into the systems, but the hydrodynamic diameter values presented a major population of ~490 nm, probably related to the formation of PL L-81 aggregates in aqueous solution. PL L-81 is a copolymer with a longer hydrophobic block, low molecular weight, and hydrophilic-lipophilic balance (HLB) value of 2, when compared to PL 407 (HLB = 22) and PL 338 (HLB = 29). The formation of binary systems with PL 407 and PL L-81 was recently described in the literature, but considering a concentration ratio (in %) of 1 : 10 (PL L-81 : PL 407) [71]. In this work, we have tested a concentration ratio (in %) of 1 : 4 (PL L-81 : PL 407), considering the incorporation of a hydrophobic drug such as levofloxacin (*P*_octanol/water_ = 4.3).

Zeta potential is a useful indicator of the surface charge of the particles. The zeta potential values were used to predict and control the stability of the formulations. The higher the zeta potential is, the more likely the formulation is to be stable because charged particles repel each other and this force can overcome the natural tendency to aggregation. The polydispersity index varied from 0.191 to 0.444 and the data obtained statistically by ANOVA had *p* > 0.05. For intravenous administration, it is desirable that the present micellar polydispersity index is lower than 0.2.

**Figure 2:**
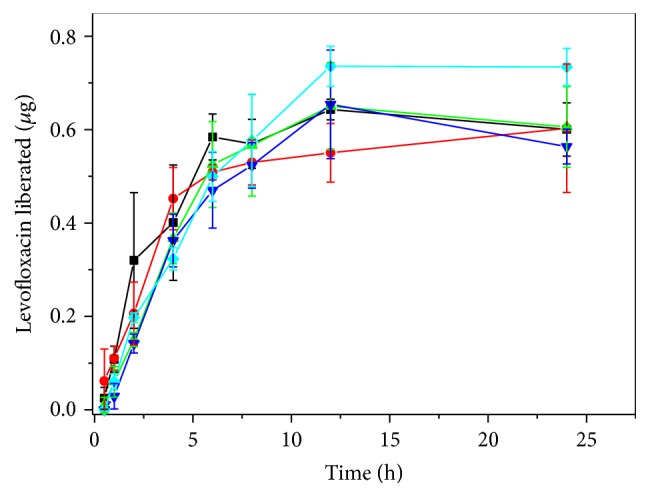
Levofloxacin liberation across Franz diffusion cells. L (-■-), F1 + L (-●-), F2 + L (-▲-), F3 + L (-▼-), and F4 + L (-◆-) were carried out in Franz diffusion cells for 24 h, and the concentration of levofloxacin permeate was calculated. The results represent the means ± SD.

The analysis of permeation study through Franz diffusion cells was shown in Figure 2 whereas the permeation profile was obtained for levofloxacin across Franz diffusion cells from the formulation studies. According to these results, the presence of poloxamers did not affect levofloxacin liberation.

### 3.1. Antimicrobial Susceptibility Studies

Nevertheless, the use of poloxamers, particularly in Formulation 1, showed an important effect in decreasing the MIC of levofloxacin. These formulations decreased the MIC in 53.3% of the strains when compared with only levofloxacin. In other strains, the result was similar to levofloxacin's. For the same strains, Formulations 2, 3, and 4 with levofloxacin decreased the MIC in 33.3%, 40%, and 40%, respectively.

These results reinforce the hypothesis of the new use of nanoformulations for controlled delivery of antibiotics in infections caused by resistant bacteria. Furthermore, according to the MIC analysis, the preferential effect of these formulations in Gram-negative strains indicates a possible interaction of poloxamer preparations with a membrane of these bacteria (as, e.g.,* Pseudomonas*,* E. coli*, and* Klebsiella* strains used in this work, Tables 2 and 4).

Fluoroquinolone-loaded polymeric micelles reduce murine fibroblast but not levofloxacin and polymeric micelles.

In analysis of cytotoxicity in NIH/3T3 murine fibroblast, the formulations using levofloxacin incorporated in Formulations 1, 2, 3, and 4 presented a low cytotoxic effect. This effect could be used in the treatment of, for example, burned skin, helping the removal of damaged tissue and increasing the penetration of the antibiotic, thus improving the treatment of possible infections.

**Figure 3:**
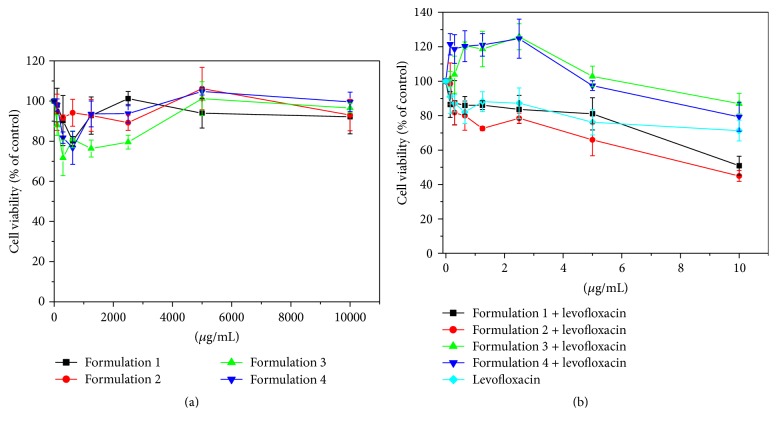
Effects of polymeric micelles and fluoroquinolone-loaded polymeric micelles on BALB/c 3T3 cell viability. BALB/c 3T3 cells were treated with different concentrations of polymeric micelles (a) and fluoroquinolone-loaded polymeric micelles (b) for 24 h, and cell viability was assessed by MTT reduction assay. The results represent the means ± SD.

From the MTT reduction assay, it was observed that polymeric micelles (Figure 3(a)) did not affect the viability of murine fibroblast (NIH/3T3), while this cell was sensitive to levofloxacin and fluoroquinolone-loaded polymeric micelles (Figure 3(b)). Formulations 1 and 2 had an IC_50_ of around 10 *μ*M; Formulations 3 and 4 and levofloxacin were much less sensitive; cell viability dropped only 13%, 19%, and 30%, respectively, in the concentration of 10 *μ*M of levofloxacin.

## 4. Conclusions

In conclusion, the possible uses of combined formulations of antibiotics and thermoregulated poloxamers have new perspectives for clinical uses, mainly in the infections in damaged skin and exposed tissues. New formulations using combined antibiotics are worth our attention for systemic or parenteral use.

## Figures and Tables

**Table 1:** Composition of polymeric nanoparticle composed of poloxamers.

Formulation	Poloxamer 1 (concentration)	Poloxamer 2 (concentration)
F1	PL 407 (8%)	PL L-81 (2%)
F2	PL 108 (8%)	PL L-81 (2%)
F3	PL 407 (8%)	—
F4	PL 108 (8%)	—

PL 407 (Pluronic F127); PL 108 (Pluronic 108); and PL L-81 (Pluronic L-81).

**Table 2:** Bacterial strain, characteristics, and origin of all bacteria studied in this work.

Strain	Characteristic	Origin
ATCC 129214	*Escherichia coli ATCC *129214	ATCC
CS1	*Escherichia coli *clinical isolation	LABIOTEC
CS2	*Escherichia coli *clinical isolation	LABIOTEC
CS3	*Escherichia coli *clinical isolation	LABIOTEC
37 JF	*Pseudomonas aeruginosa *37 JF	LEBEM
24 JF	*Pseudomonas aeruginosa *24 JF	LEBEM
76 JF	*Pseudomonas aeruginosa *76 JF	LEBEM
ATCC 27853	*Pseudomonas aeruginosa *ATCC 27853	ATCC
CS4	*Pseudomonas aeruginosa *clinical isolation	LABIOTEC
ATCC 700603	*Klebsiella pneumoniae *ATCC 700603	ATCC
CS5	*Klebsiella pneumoniae *clinical isolation	LABIOTEC
CS6	*Klebsiella pneumoniae *clinical isolation	LABIOTEC
CS7	*Klebsiella pneumoniae *clinical isolation	LABIOTEC
ATCC 25922	*Staphylococcus aureus*	ATCC
BEC	*Staphylococcus aureus *Brazilian epidemic clone	LABIOTEC

LEBEM: Special Laboratory of Bacteriology and Molecular Epidemiology, Department of Clinical, Toxicological and Bromatological Analysis, São Paulo State University, USP, Ribeirão Preto.

ATCC: American Type Culture Collection.

LABIOTEC: Biotechnology Laboratory, Campinas State University (UNICAMP), Campinas, SP, Brazil.

**Table 3:** Hydrodynamic diameter (nm), mean distribution (%), and zeta potential (mV) of micelles composed of PL 407 or PL 108 isolated or in association with PL L-81 containing levofloxacin (LVF).

Formulations	25°C			37°C		
	Hydrodynamic diameter (nm)	Mean distribution (%)	Zeta potential (mV)	Hydrodynamic diameter (nm)	Mean distribution (%)	Zeta potential (mV)
F1	5.2 ± 0.1 39.8 ± 0.9	10.5 ± 0.9 89.5 ± 0.2	−15.30 ± 1.03	21.0 ± 0.6	100	−5.45 ± 0.34
F2	5.6 ± 0.2 46.0 ± 1.5	10.7 ± 1.1 89.3 ± 1.0	−15.70 ± 1.19	22.5 ± 1.7	100	−14.02 ± 0.54
F3	6.2 ± 0.1 467.8 ± 42.1	12.7 ± 0.2 87.3 ± 0.3	−3.62 ± 1.58	482.1 ± 20.2	100	−6.63 ± 0.85
F4	37.0 ± 0.1 492.1 ± 0.1	31.7 ± 0.1 62.7 ± 0.2	−2.57 ± 1.31	495.4 ± 3.2	98.3 ± 2.3	−2.54 ± 0.64
F1-L	552.4 ± 43.9	99.2 ± 0.8	−6.03 ± 0.63	333.2 ± 2.6	89.2 ± 12	−2.54 ± 0.64
F2-L	6.2 ± 0.06 458.4 ± 19.8	12.8 ± 0.2 87.2 ± 0.2	−16.60 ± 0.43	338.3 ± 6.7	95.8 ± 3.7	−16.60 ± 0.54
F3-L	552.3 ± 44.2	98.7 ± 1.2	−2.49 ± 0.65	539.8 ± 80.6	92.2 ± 7.6	−14.40 ± 0.45
F4-L	580.1 ± 0.1	93.1 ± 5.6	−1.90 ± 0.82	587.5 ± 4.9	93.1 ± 2.4	−6.24 ± 0.65

Note: data presented as mean ± standard deviation (*n* = 3/formulation).

**Table 4:** Bacteria susceptibility to antimicrobial agent, levofloxacin, and the formulation with levofloxacin.

Strain	Levofloxacin (L)	F1 + L	F2 + L	F3 + L	F4 + L
ATCC 129214	0.15 *µ*g	0.15 *µ*g	0.15 *µ*g	0.15 *µ*g	0.15 *µ*g
CS1	10 *µ*g	5 *µ*g	10 *µ*g	5 *µ*g	5 *µ*g
CS2	10 *µ*g	10 *µ*g	10 *µ*g	10 *µ*g	10 *µ*g
CS3	0.3 *µ*g	0.15 *µ*g	0.15 *µ*g	0.3 *µ*g	0.3 *µ*g
37 JF	0.3 *µ*g	0.15 *µ*g	0.15 *µ*g	0.15 *µ*g	0.15 *µ*g
24 JF	0.5 *µ*g	0.15 *µ*g	0.3 *µ*g	0.3 *µ*g	0.3 *µ*g
76 JF	*R*	*R*	*R*	*R*	*R*
ATCC 27853	*R*	10 *µ*g	*R*	*R*	*R*
CS4	0.30 *µ*g	0.15 *µ*g	0.30 *µ*g	0.15 *µ*g	0.15 *µ*g
ATCC 700603	0.5 *µ*g	0.15 *µ*g	0.15 *µ*g	0.3 *µ*g	0.3 *µ*g
CS5	*R*	*R*	*R*	*R*	*R*
CS6	0.15 *µ*g	0.15 *µ*g	0.15 *µ*g	0.15 *µ*g	0.15 *µ*g
CS7	10 *µ*g	10 *µ*g	10 *µ*g	10 *µ*g	*R*
ATCC 25922	0.30 *µ*g	0.15 *µ*g	0.15 *µ*g	0.15 *µ*g	0.15 *µ*g
BEC	5 *µ*g	5 *µ*g	*R*	5 *µ*g	5 *µ*g

*R*: resistance MIC point.
